# Four new species of *Philoplitis* Nixon (Braconidae, Microgastrinae) with an updated key and illustrations of all described species

**DOI:** 10.3897/zookeys.841.33549

**Published:** 2019-05-03

**Authors:** A.P. Ranjith, J. Fernandez-Triana, T. Veena, D.R. Priyadarsanan, M. Nasser

**Affiliations:** 1 Insect Ecology and Ethology Laboratory, Department of Zoology, University of Calicut, Kerala, Pin: 673635, India; 2 Canadian National Collection of Insects, 960 Carling Ave., Ottawa, ON K1A 0C6, Canada; 3 Department of Zoology, Malabar Christian College, Kozhikode (Affiliated to University of Calicut), 673001, Kerala, India; 4 Ashoka Trust for Research in Ecology and the Environment, Bangalore, Karnataka, Pin: 560 064, India

**Keywords:** Afrotropical, Oriental, Microgastrinae, *Philoplitis*, taxonomic revision

## Abstract

The Microgastrinae genus *Philoplitis* Nixon is revised and four new species are described: *P.keralensis***sp. n.** and *P.trifoveatus***sp. n.** authored by Ranjith & Fernandez-Triana, and *P.dzangasangha***sp. n.** and *P.margalla***sp. n.** authored by Fernandez-Triana & Ranjith. A key to all nine known species is provided. *Philoplitisadustipalpus* Ahmad is redescribed and illustrated. Additional specimen records are presented, and the diagnostic value of some morphological characters previously used is discussed. Based on the very few specimens available for study in collections, *Philoplitis* seems to be restricted to the Old World tropics (Afrotropical and Oriental regions), with most known species found in the Oriental region. The first DNA barcodes for the genus are presented. No host data is currently available, but for one species a mass of five wasp cocoons was found and is illustrated for the first time.

## Introduction

Microgastrinae is one of the most diverse and globally ubiquitous subfamilies of braconid parasitoid wasps, commonly encountered as pupae or prepupae encased in white silk cocoons on or near the dead or dying bodies of their host caterpillars. Species are the koinobiont endo-larval parasitoids of Lepidoptera (Quicke 2015). More than 100 species in this group have been used or investigated worldwide in the biological control of lepidopteran pests, and this total is likely to raise (Wharton et al. 1997).

Mason (1981) estimated that worldwide Microgastrinae comprises between 5,000 and 10,000 species; at present Microgastrinae contains 81 extant genera and 2,700+ extant species (Yu et al. 2016, Fernandez-Triana and Boudreault 2018). Rodriguez et al. (2013) estimated the species richness of microgastrines to be 8–10 times that of the ~2,000 species described at that time. The microgastrine fauna of India contains 231 species recorded within 21 genera so far (Gupta and Fernandez-Triana 2014). Recent studies show that there are many new distribution records like *Alloplitis* Nixon (Ranjith, unpublished) and many more species in both diverse and rare genera (Veena et al. 2014, Ranjith et al. 2015).

The genus *Philoplitis* Nixon was erected by Nixon (1965) and the species can be easily diagnosed by the large scutellum, which is prolonged posteriorly above the propodeum (Mason 1981). Fernandez-Triana and Goulet (2009) revised the genera, described three new species, and provided a key to all known species. The distribution of *Philoplitis* seems to be restricted to the Afrotropical and Oriental regions (Fernandez-Triana and Goulet 2009, current study). The hosts of *Philoplitis* species are still unknown.

Here we describe four new species from the Old World tropics, redescribe *Philoplitisadustipalpus* Ahmad, present additional records for some species, and discuss the diagnostic value of some morphological characters previously used.

## Materials and methods

We examined the specimens from various type repositories including the Centre for Biodiversity Genomics, University of Guelph, Canada (**BIOUG**), the Canadian National Collection of insects, Canada (**CNC**), the National Museum of Natural History, USA (**NMNH**), and the collection of the Zoology Department, Aligarh Muslim University, Uttar Pradesh, India (**ZDAMU**). Morphological terms and wing venation designations follow Huber and Sharkey (1993), Sharkey and Wharton (1997), Karlsson and Ronquist (2012), and Fernandez-Triana et al. (2014). The abbreviations F2 and F15 refer to antennal flagellomeres 2 and 15; T1, T2, and T3 are used for metasomal mediotergites 1, 2 and 3; and L and W refer to length and width, respectively. Calculation of wing vein ratios follows Fernandez-Triana and Goulet (2009). The terms for integument sculpture follow Harris (1979). Primary types are deposited in Department of Zoology, University of Calicut, Kerala, India (**DZUC**) and the CNC.

Indian specimens studied by the first author were treated with Hexamethyldisilazane (HMDS) in order to prevent collapsing during mounting. Specimens were imaged using an Leica M205A microscope with automated multiple image capture at preset focal levels using an Leica DFC 500 camera, and image combination using the Leica Application Suite (V4.7) image processing system. All images were edited using Photoshop CS8 (Version 6.1) (Adobe Inc.) for the removal of image artifacts and standardizing the background colour.

All specimens studied from BIOUG and CNC material (African and Oriental regions) were critical point dried, and imaged with a Keyence VHX-1000 Digital Microscope, using lens with a range of 10–130×. Multiple images were taken of a structure through the focal plane and then combined to produce a single in-focus image using the software associated with the Keyence System. Images were corrected using Adobe Photoshop CS4, and plates were prepared using Microsoft PowerPoint 2010 and later saved as .tiff files.

A distribution map of all known species of *Philoplitis* was made using Simplemappr (https://www.simplemappr.net).

## Results

With the four new species described below, the total number of known *Philoplitis* almost doubles, from five (Fernandez-Triana and Goulet 2009) to nine in this paper. It is likely that a few more species are found in the future, especially when the fauna of the Afrotropical and Oriental regions is more comprehensively collected and studied. Still, this genus is not likely to be very specious, and the specimens seem to be very rarely collected. Indeed, most of the species are known from one or very few specimens in collections (Table 1), despite of hundreds of microgastrinae specimens collected in some of the localities where *Philoplitis* specimens have been found so far.

**Table 1.** Described species of *Philoplitis* with their known geographical distribution (by biogeographical region and country), and the number of specimens in collections. Data from Nixon (1965), Mason (1981), He (1983), Ahmad et al. (2005), Fernandez-Triana and Goulet (2009), and present paper.

***Philoplitis* species**	**Known distribution as Biogeographical region: Countries**	**Number of known specimens**
*P.adustipalpus* Ahmad, 2005	Oriental: India	2 female
*P.coniferens* Nixon, 1965	Oriental: Philippines, China	12+ female and male
*P.dzangasangha* Fernandez-Triana & Ranjith, sp. n.	Afrotropical: Central African Republic, Republic of Congo	5 male, 1 female
*P.keralensis* Ranjith & Fernandez-Triana, sp. n.	Oriental: India	1 female, 1 male
*P.margalla* Fernandez-Triana & Ranjith, sp. n.	Oriental: Pakistan	2 female, 1 male
*P.masneri* Fernandez-Triana & Goulet, 2009	Afrotropical: Kenya	1 male
*P.punctatus* Fernandez-Triana & Goulet, 2009	Oriental: Thailand	1 female, 4 male
*P.striatus* Fernandez-Triana & Goulet, 2009	Oriental: India, Sri Lanka	4 female, 3 male
*P.trifoveatus* Ranjith & Fernandez-Triana, sp. n.	Oriental: India	1 female

**Figure 1.**
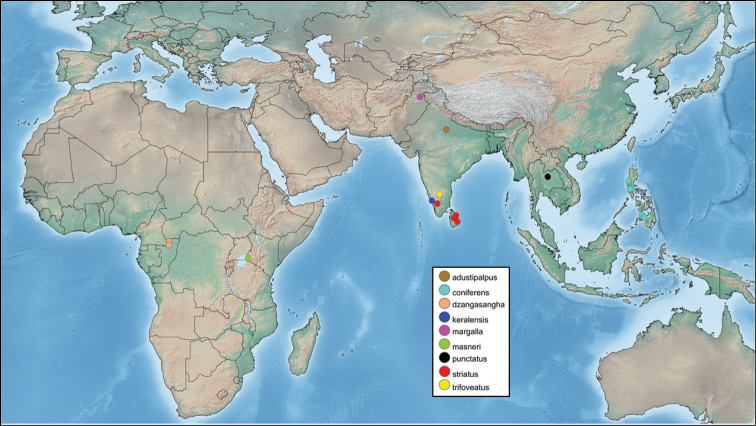
Distribution of known species of *Philoplitis*.

Based on current data, the genus seems to be restricted to the Old World tropics, with the majority of the species being found in the Oriental region and two species in the Afrotropics (Fig. 1). No host data is known at present but for one species, *P.keralensis* Ranjith & Fernandez-Triana, sp. n., a mass of five wasp cocoons was found associated with an unidentified lepidopteran larvae and is here illustrated for the first time (Fig. 8). We suspect other species of *Philoplitis* may be gregarious as well.

Seven sequences (DNA barcodes) are available in the Barcode of Life Data System (BOLD), representing three of the nine described species (Suppl. material 1, Supplementary Appendix 1). Those sequences are grouped in four Barcode Index Numbers (for details on the BIN system see Ratnasingham and Hebert 2013). The current BINs representing *Philoplitis* are: BOLD:AAW0954, BOLD:AAZ9051, BOLD:ACA6591, and BOLD:ACP5254 (http://www.boldsystems.org/index.php/Public_BINSearch?query=Philoplitis&searchBIN=Search+BINs). The DNA barcodes of *Philoplitis* are very distinct, and cluster fairly separated from 35,000+ sequences of other microgastrines available in BOLD.

Future work should focus on better morphological characterization of the species, as the majority have been described from and/or are currently known from very few specimens (Table 1). Fernandez-Triana and Goulet (2009) put too much emphasis on the relative proportions of the fore wing veins near the areolet, but those proportions may be found to vary when more specimens become available. We have found that color of palpi and metatibia spurs, sculpture of head near occipital carina, and metafemur L/W ratio seem to have more diagnostic value than vein proportions in the fore wing. But we also caution that if/when more specimens become available, the value of those characters may also need to be reassessed.

### Key to *Philoplitis* species

**Table:** 

1	Metatibial spurs dark brown to black in both male and female specimens (Fig. 11A); scutellar disc (Figs 11D–F) comparatively shorter (length 1.00–1.10 × its width at anterior margin); fore wing with vein r from slightly less to about same length than vein r-m (Fig. 11C) [Thailand]	***Philoplitispunctatus* Fernandez-Triana & Goulet, 2009**
–	Metatibial spurs white, yellow or yellowish-brown in female specimens (and males of all species but one) (Figs 2A, 3D, 5A, 5B, 6A, 9A, 10A, 12A, 13A, 14A); scutellar disc comparatively longer (length at least 1.20 × its width) (Figs 2D, 4A, 5F, 6D, 9G, 10B, 12G, 13B, 14D, 15B); fore wing with vein r much longer than vein r-m (Figs 5B, 7B, 12A, 12C, 13A, 14A)	**2**
2	Anteromesoscutum with coarse punctures along anterior and lateral margins (Fig. 9G); male specimens with metatibial spurs dark brown to black [Pakistan]	***Philoplitismargalla* Fernandez-Triana & Ranjith, sp. n.**
–	Anteromesoscutum without coarse punctures along anterior and lateral margins, but sometimes with impressed sculpture postero-laterally (Figs 2D, 3B, 4A, 5F, 6D, 7A, 10B, 14D); male specimens with metatibial spurs white or yellow	**3**
3	Maxillary and labial palpi entirely yellow (Fig. 5E); pro- and mesocoxae yellow (Figs 5B, 5E); antennal flagellomeres yellow-brown (Fig. 5A); T2 with raised, median, polished field (Fig. 5D); impressed area behind posterior ocelli (Fig. 5F) mostly smooth and relatively very wide, as wide as distance between two posterior ocelli [Central African Republic]	***Philoplitisdzangasangha* Fernandez-Triana & Ranjith, sp. n.**
–	Maxillary and labial palpi partially (Figs 10D, 12B) to entirely reddish-brown to dark brown (Figs 2B, 2C, 3A, 6B, 6C, 14B, 15A, 15B); all coxae either reddish, dark brown or black (Figs 2F, 3A, 3D, 6A, 6C, 10A, 12A, 14A, 15A); antennal flagellomeres dark brown to black; T2 without raised median field (Figs 3C, 4B, 6F, 10C, 15C), if (rarely) median field is visible, then it is striated (Fig. 13D); impressed area behind posterior ocelli partially to entirely striate and relatively less wide, narrower than distance between two posterior ocelli (Figs 10E, 12G, 16A–D)	**4**
4	Metatibial spurs white; scutellum slightly truncate apically (Fig. 10E); notauli comparatively less impressed (Fig. 10B); T2 comparatively less transverse, its medial length 0.80 × its width at posterior margin (Fig. 10C) [Kenya]	***Philoplitismasneri* Fernandez-Triana & Goulet, 2009**
–	Metatibial spurs yellow or yellowish-brown; scutellum longer and not truncate (Figs 2D, 4A, 6D, 13B, 14D, 15B); notauli comparatively deeply impressed (Figs 4A, 6D, 12D, 13B, 14D); T2 comparatively more transverse, its medial length less than 0.50 × its width at posterior margin (Figs 3C, 4B, 6E, 6F, 13D, 15C)	**5**
5	T2 dark brown, transversely striate medially and longitudinally striate latero-apically (Figs 12F, 13D); T1 length less than 1.80 × its apical width [India, Sri Lanka]	***Philoplitisstriatus* Fernandez-Triana & Goulet, 2009**
–	T2 yellowish-white or yellowish brown and almost completely smooth (Figs 3C, 4B, 6E, 6F, 15C); T1 length more than 2.00 × its apical width	**6**
6	Body color mostly reddish-brown (Figs 2, 3); T1 length 2.20 × its apical width; T2 with medial length more than 0.40 × its width at apex; palpi usually brown or light brown with apical segments yellowish	**7**
–	Body color mostly black (Figs 6, 14, 15); T1 length more than 2.20 × its apical width; T2 with medial length less than 0.40 × its width at apex; palpi brown	**8**
7	F2 L/W 2.30 × and F15 L/W 2.70 ×; maxillary palps 3–5 yellowish-white; notauli deeply impressed (Fig. 4A); tarsal claw with 2–3 teeth; T1 without shallow longitudinal groove, apically orange-yellow (Fig. 4B); T2 with median zone outlined by shallow and quite divergent grooves (Fig. 4B) [Philippines and southeastern China]	***Philoplitisconiferens* Nixon, 1965**
–	F2 L/W 3.00 × and F15 L/W 1.90 ×; maxillary palps 3–5 dark brown (Fig. 3A); notauli faintly impressed (Figs 2D, 3A); tarsal claw with one tooth; T1 with distinct shallow longitudinal groove extending beyond middle of the tergite, apically yellow (Fig. 2F); T2 without median zone outlined by shallow and quite divergent grooves (Fig. 3C) [India]	***Philoplitisadustipalpus* Ahmad, 2005**
8	Occiput medially with three pits right above and before occipital carina (Fig. 16 C); mesopleuron impressed medially (Fig. 14C); fore wing infuscated only beneath pterostigma (Figs 14A, 15C); fore wing with length of vein r 1.00 × length of vein 3RSa, and length of vein 3RSa 1.10 × length of vein 2M (Figs 14A, 15C); tarsal claw with one tooth; metafemur length 3.40 × its maximum width; T1 with longitudinal groove extending beyond half of tergite (Fig. 14F); T2 orange-brown (Fig. 15C) [India]	***Philoplitistrifoveatus* Ranjith & Fernandez-Triana, sp. n.**
–	Occiput medially with one pit right above and before occipital carina (Fig. 16B); mesopleuron not impressed medially (Fig. 6C); fore wing infuscated throughout beneath pterostigma (Figs 6A, 7B); fore wing with length of vein r 1.60 × length of vein 3RSa, and length of vein 3RSa 0.47 × length of vein 2M (Figs 6A, 7B); tarsal claw with two teeth; metafemur length 3.70 × its maximum width; T1 with longitudinal groove not extending beyond half of tergite (Fig. 6E); T2 yellow (Figs 6E, 6F) [India]	***Philoplitiskeralensis* Ranjith & Fernandez-Triana, sp. n.**

## Species descriptions and notes

### Philoplitis adustipalpus

Taxon classificationAnimaliaHymenopteraBraconidae

Ahmad, 2005

Figs 2 , 3 , 16A

Philoplitis adustipalpus Ahmad, 2005: 1736, original description.

#### Material examined.

**Holotype**: Female, INDIA (ZDAMU). Holotype locality: India, Uttar Pradesh, Etawah, 13.iv.2002, M Shamim leg.

#### Diagnosis.

*Philoplitisadustipalpus* is similar to *P.coniferens* Nixon by its body colour, but it can be distinguished by having mesopleuron mostly rugose (punctate in *P.coniferens*), and metatibial spurs whitish yellow (orange-yellow in *P.coniferens*).

#### Redescription.

Head distinctly rugose, frons transversely striated. Occipital carina strongly defined and crenulate. Moderately smooth and shiny area centrally between posterior ocelli and occipital carina. Antennae long, L of F2 3.00 × its W, L of F15 1.90 × its W. Mesosoma mostly covered with silver setae. Anteromesoscutum coarsely rugose. Notauli faintly impressed with impressed postero-lateral area above tegula. Scutellar disc L/W ratio 1.20 ×, and its length 0.80 × that of anteromesoscutum. Mesopleuron rugose anteriorly, smooth to sparsely punctate medially, rugose posteriorly with a median, smooth area centrally. Metapleuron rugose. Propodeum rugose with complete, medial longitudinal carina, lateral carinae forming crenulations. Tarsal claws with one tooth and with arolium subequal to claw length. Metafemur L 3.47 × its maximum width. Inner spur of metatibia 0.39 × L of first metatarsomere. T1 finely striate, slightly emarginate medially with smooth triangular area medio-apically, T1 with shallow, median longitudinal groove extending beyond half of tergite length, T1 L 2.20 × its W at posterior margin. T2 smooth, medial zone outlined by divergent carinae on either side, T2 medial L 0.47× its W at posterior margin. T3+ smooth, sparsely setose.

**Figure 2.**
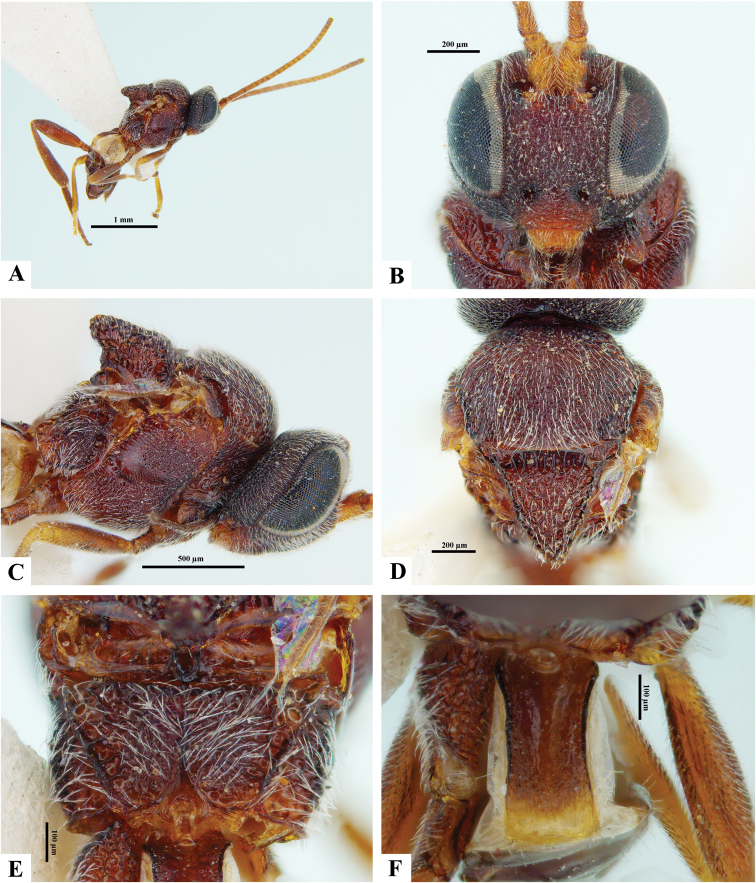
*Philoplitisadustipalpus*, female holotype **A** habitus, lateral view **B** head, frontal view **C** head and mesosoma, lateral view **D** mesosoma, dorsal view **E** propodeum, dorsal view **F** metasomal tergite I, dorsal view.

**Figure 3.**
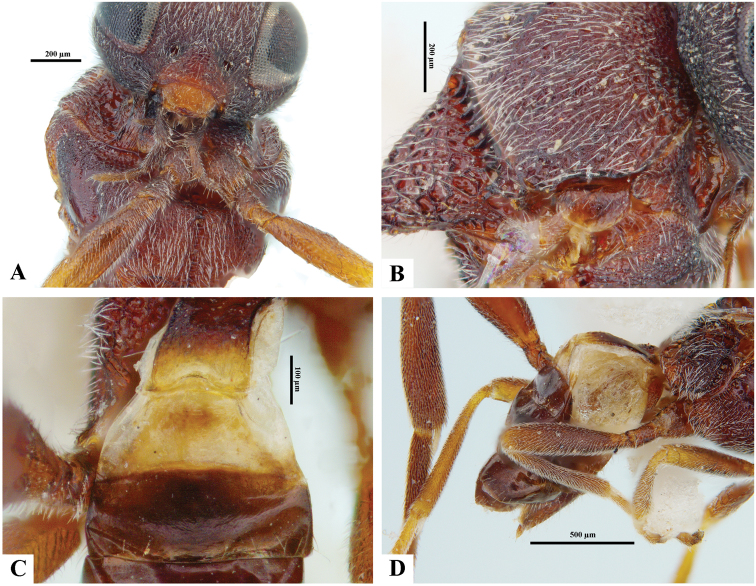
*Philoplitisadustipalpus*, female holotype **A** head and mesosoma, ventral view **B** mesosoma, oblique view **C** metasomal tergites 2, 3, dorsal view **D** metasoma, lateral view.

#### Colour.

Head reddish brown, clypeus reddish brown, mandibles brown, palpi brown, antennae yellowish brown, gena laterally black, mesosoma reddish brown with silver pubescence, tegula brown, procoxa and protrochanter brown, profemur brown basal half apically yellow, protibia and protarsomeres yellow, mid leg and hind leg brown, metatibial spurs whitish yellow, metasoma dark brown, T1 black laterally, apically yellow, T2 yellow with light brownish area medially, laterotergites 1 and 2 yellowish white.

#### Male.

Unknown.

#### Distribution.

India (Uttar Pradesh).

### Philoplitis coniferens

Taxon classificationAnimaliaHymenopteraBraconidae

Nixon, 1965

Fig. 4

Philoplitis coniferens Nixon, 1965: 267, original description.

#### Notes.

This species was redescribed and diagnosed by Fernandez-Triana and Goulet (2009). Here we report additional specimens (2 females, 4 males) from a different locality in Philippines, Cavite, Maragondon, 14°15.97'N 120°42.71'E, iv-v.2011, Malaise trap, H Ngo leg. (CNC).

**Figure 4.**
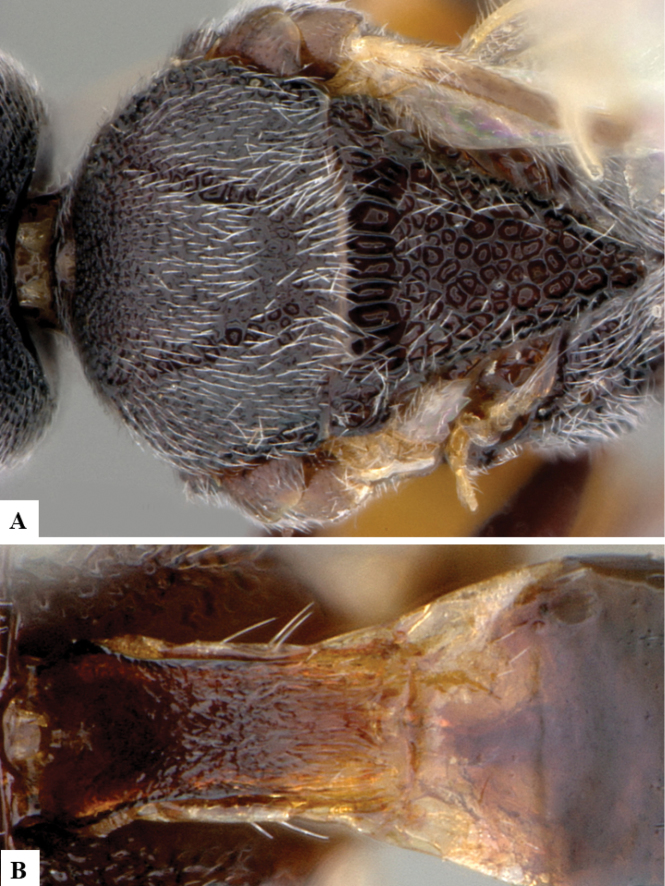
*Philoplitisconiferens*, female **A** mesosoma, dorsal view **B** metasomal tergites 1–3, dorsal view.

### Philoplitis dzangasangha

Taxon classificationAnimaliaHymenopteraBraconidae

Fernandez-Triana & Ranjith sp. n.

http://zoobank.org/3EFD60B4-54DC-40B7-9E4D-60E9A4D0DABF

Fig. 5

#### Holotype.

Male, CENTRAL AFRICAN REPUBLIC (CNC). Holotype locality: Central African Republic, Dzanga-Sangha Reserve, 2°55'N 16°15'E, 320 m, 5-16.vii.1998, Malaise trap, J Carpenter & J Wensel leg.

#### Paratypes.

Four males, REPUBLIC OF THE CONGO (CNC), Sangha province, Bomassa, 2.2105 16.1929, 365 m, mixed forest with *Maranthacae* sp., 30.x.2012, Cysquet, Darlina & Lyenguet leg.

#### Non type material examined.

One female, same locality and colleting date than paratypes.

#### Diagnosis.

*Philoplitisdzangasangha* sp. n. differs from all other species by its generally lighter coloration with palpi, pro- and mesocoxae yellow, and flagellomeres light brown.

#### Description.

Head distinctly rugose. Frons transversely striate, with a median, longitudinal carina. Occipital carina strongly defined and crenulate. Smooth and shiny area centrally between posterior ocelli and occipital carina. Antennae longer than body length, L of F2 2.50 × its W, L of F15 3.00× its W. Mesosoma mostly covered with silver setae. Anteromesoscutum coarsely rugose. Notauli deeply impressed, and with impressed postero-lateral area above tegula. Scutellar disc coarsely rugose, more or less straight in lateral view. Scutellar disc L/W ratio 1.20×, and its L 0.80× that of anteromesoscutum. Mesopleuron mostly rugose or striate, but with median, smooth area centrally. Metapleuron rugose. Propodeum rugose with complete mid longitudinal carina. Fore wing ratios, r/3RSa: 2.00 ×; r/r-m: 2.80 ×; r/2RS: 1.00 ×; 3RSa/2M: 1.10 ×; 2RS/2M: 0.90 ×; r-m/2M: 0.60 ×; r/(r-2M): 0.51 ×; height of second sub marginal cell/(r-2M): 0.33 ×. Tarsal claws with one tooth and with arolium longer than claw length. Metafemur L 3.20 × its maximum width, inner spur of metatibia 0.54 × L of first metatarsomere. T1 mostly sculptured on posterior 0.7 (anterior 0.3 mostly smooth), T1 with shallow, median longitudinal groove extending half of tergite length, T1 L 3.00 × its width at posterior margin. T2 smooth, subtriangular, poorly defined by divergent grooves (grooves only distinct on anterior half of T2 length), T2 medial L 0.50 × its W at posterior margin (but that value is approximate as W at posterior margin is not clearly defined by the grooves). T3+ smooth.

**Figure 5.**
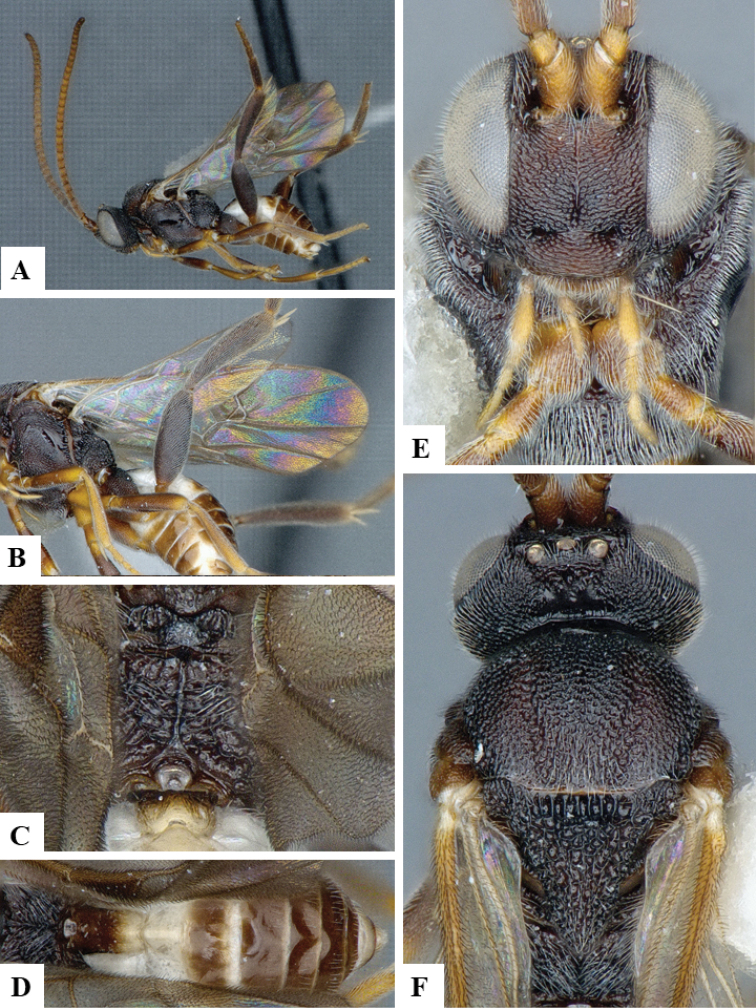
*Philoplitisdzangasangha*, male holotype **A** habitus, lateral view **B** wings and part of meso- and metasomae, lateral view **C** propodeum (partially), dorsal view **D** metasoma, dorsal view **E** head, frontal view **F** head and mesosoma, dorsal view.

#### Colour.

Head and mesosoma dark brown to black; scape and pedicel yellow; flagellomeres light brown; palpi yellow; pro- and mesocoxae yellow; pro- and mesofemorae and protibia yellow, mesotibia partially brown and partially yellow; hind legs mostly dark brown (except for trochanter and trochantellus yellow); metatibial spurs light yellow; most wing veins light yellow to transparent, except for pterostigma surrounding veins (r, 2RS, 3RSa, r-m, 2M, R1) which are brown; area beneath pterostigma very light brown, slightly darker than rest of wing and forming a cloud that extends to vein 2M; metasoma dorsally mostly brown, except for posterior 0.2 of T1 and entire T2 which are yellow-white; laterotergites 1–3 yellowish white, rest mostly brown.

#### Female.

Unknown, but see Notes below.

#### Distribution.

Central Africa Republic, Dzanga Sangha Reserve.

#### Etymology.

Named after the type locality, part of the important Dzanga Sangha Protected Area Complex, as recognition of the value that complex has to protect the biodiversity of central Africa.

#### Notes.

Among the specimens we studied there was a female collected on the same place and date than the paratypes; however, some morphological characters are different. With so few specimens available it is not possible at the time to conclude if the female belongs to the same species or a different one. For the time being we prefer to keep within the species, although we did not include it as a paratype.

### Philoplitis keralensis

Taxon classificationAnimaliaHymenopteraBraconidae

Ranjith & Fernandez-Triana sp. n.

http://zoobank.org/E075CF20-808E-4E98-AAB0-FB7AA5AADAFD

**Figure 6.**
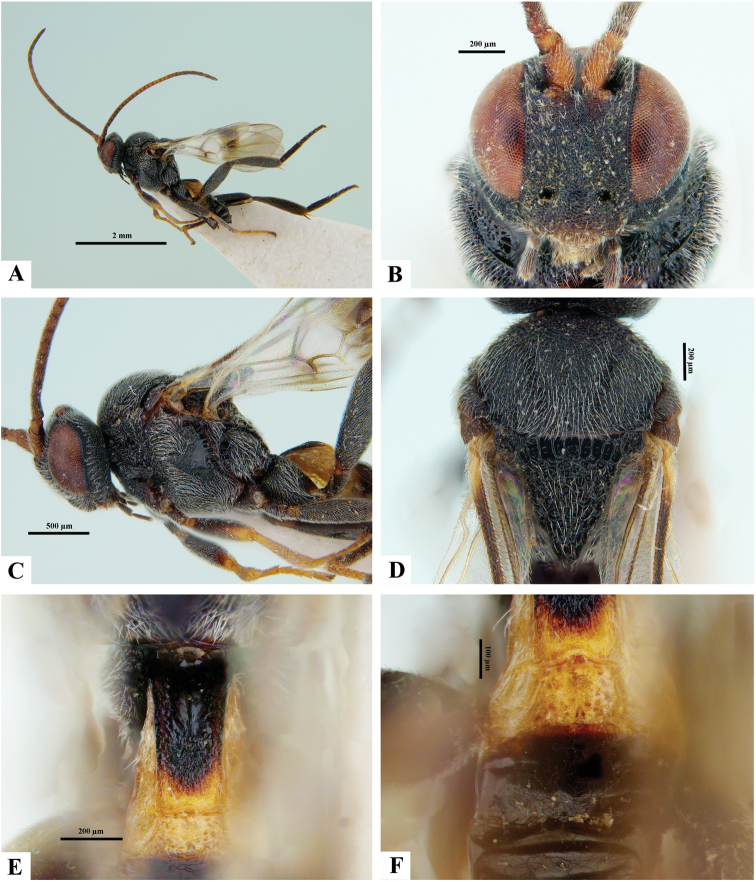
*Philoplitiskeralensis*, female holotype **A** habitus, lateral view **B** head, frontal view **C** head and mesosoma, lateral view **D** mesosoma, dorsal view **E** metasomal tergite I, dorsal view **F** metasomal tergites 2 and 3, dorsal view.

**Figure 7.**
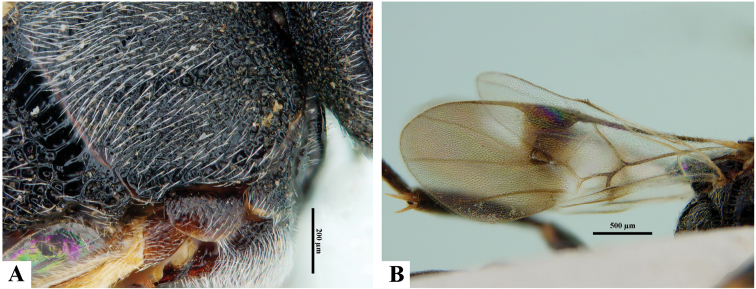
*Philoplitiskeralensis*, female holotype **A** mesoscutum (in part), oblique view **B** wings.

**Figure 8.**
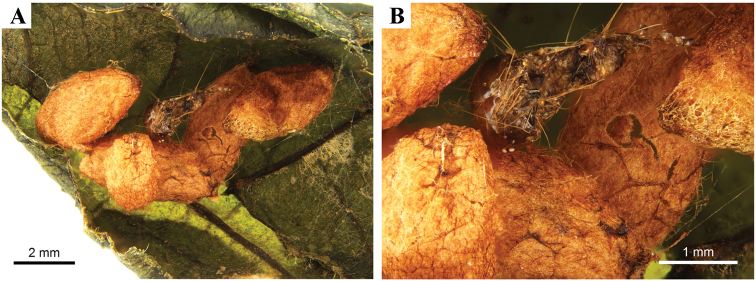
*Philoplitiskeralensis*, cocoon mass.

Figs 6 , 7 , 8 , 16B

#### Holotype.

Female, INDIA (DZUC). Holotype locality: Kerala, Kozhikode, Janakikkadu, 31.v.2013, T Veena leg.

#### Paratype.

One male, same data than holotype, except 21.v.2013, AP Ranjith leg.

#### Diagnosis.

*Philoplitiskeralensis* sp. n. is similar to *P.trifoveatus* sp. n., but it can be distinguished from the latter by having different ratios of fore wing veins 2RS/2M 1.13 × (1.30 × in *P.trifoveatus* sp. n.), eyes dark brown (eyes dark yellow in *P.trifoveatus* sp. n.), and fore wing with a small, brownish patch beneath vein 1-CU1 (without small brownish patch beneath 1-CU1 in *P.trifoveatus* sp. n.).

#### Description.

Head distinctly rugoso-punctate. Frons transversely striate. Occipital carina strongly defined and crenulated. Area centrally between posterior ocelli and occipital carina mostly coarsely sculptured, with only small, shiny spot right above the occipital carina. Antennae longer than body length, L of F2 3.09 × its W, L of F15 1.94 × its W. Mesosoma mostly covered with silver setae. Anteromesoscutum rugose near notauli, punctate laterally. Notauli impressed, and with impressed surface postero-laterally above the tegula. Scutellar disc coarsely rugose, apex pointing downward in lateral view. Scutellar disc L/W ratio 1.27 ×, and its L 0.90 × that of anteromesoscutum. Mesopleuron mostly rugoso-punctate, but with medial smooth area centrally. Metapleuron rugose. Propodeum rugose, with complete medial longitudinal carina. Fore wing ratios: r/3RSa: 1.60 ×; r/r-m: 2.11 ×; r/2RS: 0.66 ×; 3RSa/2M: 0.47 ×; 2RS/2M: 1.13 ×; r-m/2M: 0.35 ×; r/(r-2M): 0.46 ×; height of second submarginal cell/(r-2M): 0.37 ×. Tarsal claws with two teeth and with arolium subequal to claw length. Metafemur L 3.72 × its maximum width, inner spur of metatibia 0.52 × L of first metatarsomere. T1 rugose, smooth apically, T1 with shallow, median longitudinal groove extending half of tergite length, T1 L 2.42× its W at posterior margin. T2 smooth, broad, with middle zone outlined by shallow convergent grooves that form a transversely striated triangle, with carinae markedly divergent, smooth, T2 medial L 0.36× its W at posterior margin. T3+ smooth.

#### Colour.

Head and mesosoma black, scape and pedicel reddish brown, flagellomeres brown, ocelli brown, profemur mostly brown, yellow basally and apically, protibiae and protarsomeres dark brown, mesofemur and metatibia black, mesotarsomeres yellowish brown, hind leg dark brown, metatibial spurs yellowish brown, wing veins and pterostigma brown, except for junction of veins 2CU1, m-cu and 3CU1 which is white, fore wing vein 1SR-M white, with a brownish cloud beneath pterostigma that extends to the posterior margin of the wing, small brownish patch beneath 1-CU1, fore wing faintly infuscated apically. T1 apically and T2 entirely yellow. Laterotergites 1–3 yellowish-white.

#### Male.

Same as female.

#### Host.

Gregarious parasitoid of an unidentified lepidopteran larva (Fig. 8).

#### Distribution.

India (Kerala).

#### Etymology.

This new species is named after the Indian state where type locality is found.

### Philoplitis margalla

Taxon classificationAnimaliaHymenopteraBraconidae

Fernandez-Triana & Ranjith sp. n.

http://zoobank.org/C04D0DC1-E8C7-45A6-BD51-CA4CF0C4F44D

Fig. 9

#### Holotype.

Female, PAKISTAN (CNC). Holotype locality: Islamabad, Pakistan Museum of Natural History, Shakar Parian, 33°41'9.0"N 73°4'33.60"E, 18.iv.2012, Malaise trap, M Rafique leg. Voucher code: BIOUG02373-G10. Secondary voucher code: CNC469935.

#### Paratypes.

One female and one male (BIOUG, CNC). Same locality than holotype but collecting dates 5.xi.2012 and 29.xi.2012 and M Rafique & Q Abbas leg. Voucher codes: BIOUG15337-B11, BIOUG15345-A08.

#### Diagnosis.

*Philoplitismargalla* sp. n. is the only known species of *Philoplitis* with sexual dimorphism in the color of the metatibial spurs, which is light yellow in females, dark brown in males.

#### Description.

Head distinctly rugose. Frons transversely striate, without median, longitudinal carina. Occipital carina strongly defined and crenulate. Area centrally between posterior ocelli and occipital carina mostly transversally striate, with only very small, shiny spot right above the occipital carina. Antennae longer about same length as body length, L of F2 2.80 × its W, L of F15 2.40 × its W. Mesosoma mostly covered by silver setae. Anteromesoscutum coarsely rugose. Notauli deeply impressed, and with impressed postero-lateral area above tegula. Scutellar disc coarsely rugose, slightly downward apically, in lateral view. Scutellar disc L/W ratio 1.2 ×, and its L 0.86 × that of mesoscutum. Mesopleuron mostly rugose or striate, but with median, smooth area centrally. Metapleuron rugose. Propodeum rugose with complete medial longitudinal carina. Fore wing ratios, r/3RSa: 1.11 ×; r/r-m: 3.33 ×; r/2RS: 0.83 ×; 3RSa/2M: 0.90 ×; 2RS/2M: 1.20 ×; r-m/2M: 0.33 ×; r/(r-2M): 0.44 ×; height of second sub marginal cell/(r-2M): 0.44 ×. Tarsal claws simple and with arolium longer than claw length. Metafemur L 3.50 × its maximum W, inner spur of metatibia 0.50 × L of first metatarsomere. T1 fully sculptured, T1 with excavation on anterior 0.2–0.3, then a median longitudinal groove extending to half of tergite length, T1 L 2.50 × its W at posterior margin. T2 smooth, trapezoidal, relatively well defined by divergent grooves (but grooves only clearly distinct on anterior half of T2), T2 median L 0.50 × its W at posterior margin. T3+ smooth.

**Figure 9.**
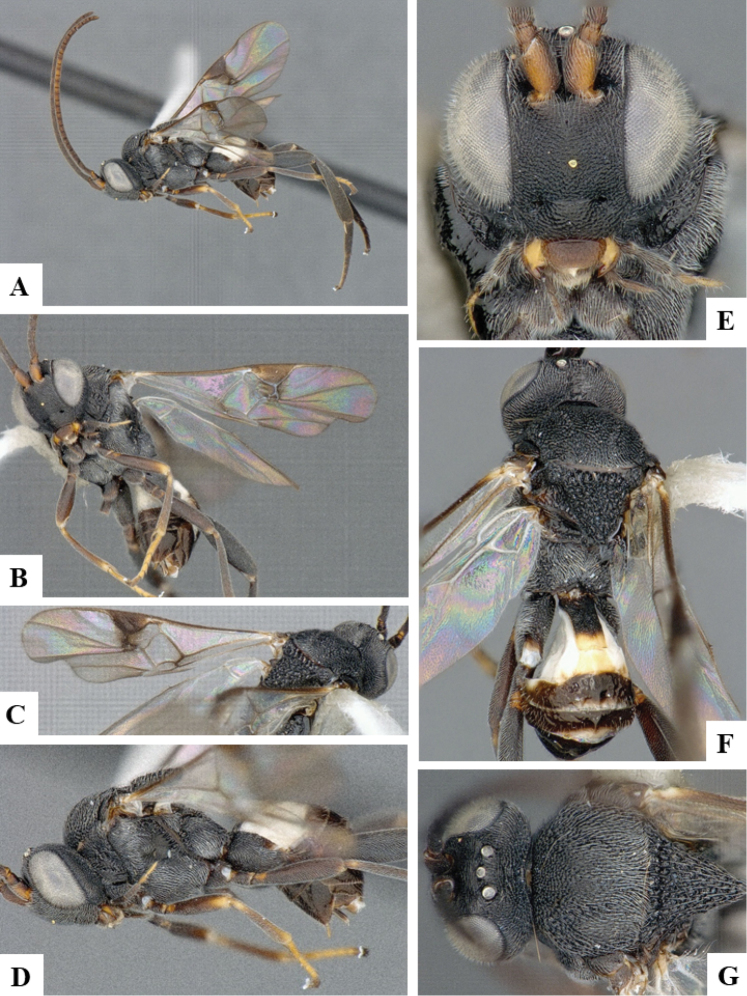
*Philoplitismargalla*, female holotype **A** habitus, lateral view **B** body and wings (partially), ventro-lateral view **C** wings **D** body, lateral view **E** head, frontal view **F** body, dorsal view **G** head and mesosoma, dorsal view.

#### Colour.

Head and mesosoma black; scape and pedicel orange-yellow; flagellomeres brown to light brown; palpi mostly dark brown to black (only two apical maxillary palpi yellow); all coxae, metafemur, metatibia and metatarsus black; pro- and mesofemorae and pro- and metatibiae mostly dark brown (at most with small yellow spot apically); pro- and mesotarsi yellow; metatibial spurs light yellow; most wing veins and pterostigma brown to light yellow; area beneath stigma light brown, clearly darker than rest of wing which is hyaline; metasoma dorsally mostly dark brown, except for T1 black on anterior 0.8, and for posterior 0.2 of T1 and entire T2 yellow-white; laterotergites 1-3 white, rest dark brown.

#### Male.

As female, but with darker body color: palpi, scape, pedicel, most of legs (including metatibial spurs) dark brown to black; metasoma dorsally and laterally darker.

#### Distribution.

Pakistan, Islamabad, Margalla Hills National Park.

#### Etymology.

The name, to be treated as a name in apposition, refers to the Margalla Hills National Park, a 12,600+ ha protected area in Islamabad Capital Territory, and where the type locality (Shakarparian Park) is located. Despite its small size, the fauna and flora of the Margalla Hills are quite diverse and mostly tropical and constitute a transitional zone between the high mountains to the north and plain areas to the south, providing a corridor for many Himalayan species to disperse south.

### Philoplitis masneri

Taxon classificationAnimaliaHymenopteraBraconidae

Fernandez-Triana & Goulet, 2009

Fig. 10

Philoplitis masneri Fernandez-Triana & Goulet, 2009: 292, figs 1, 4, 7, 10, 14 (original description).

#### Notes.

This species was described and diagnosed by Fernandez-Triana and Goulet (2009). Here we only provide new images of the species.

**Figure 10.**
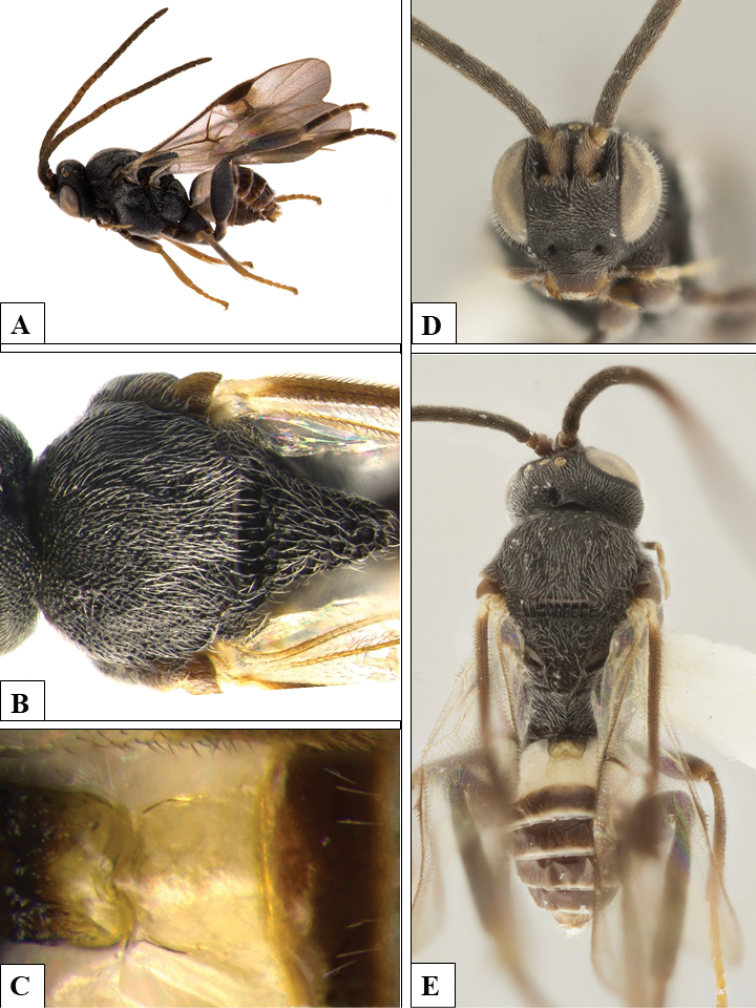
*Philoplitismasneri*, male holotype **A** habitus, lateral view **B** mesosoma, dorsal view **C** metasomal tergites 1-3 (partially), dorsal view **D** head, frontal view **E** body, dorsal view.

### Philoplitis punctatus

Taxon classificationAnimaliaHymenopteraBraconidae

Fernandez-Triana & Goulet, 2009

Fig. 11

Philoplitis punctatus Fernandez-Triana & Goulet, 2009: 293, figs 5, 8, 11, 15 (original description).

#### Notes.

This species was described and diagnosed by Fernandez-Triana and Goulet (2009). Here we only provide new images of the species, including the first illustrations of a female specimen.

**Figure 11.**
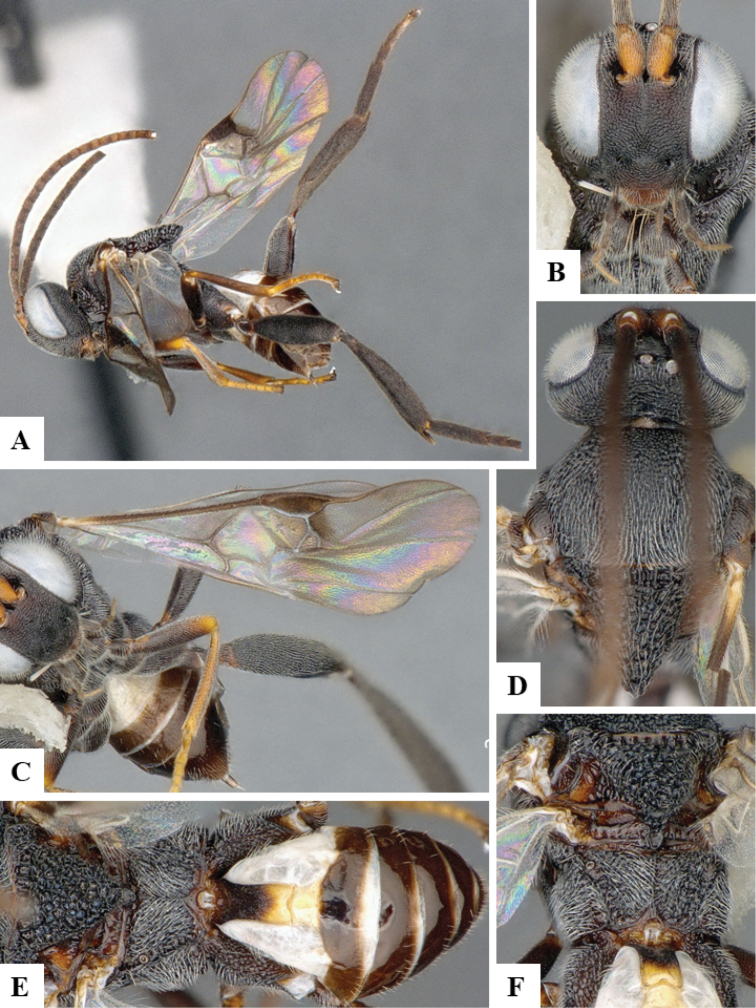
*Philoplitispunctatus*, female **A** habitus, lateral view **B** head, frontal view **C** wings and body (partially) ventral view **D** head and mesosoma, dorsal view **E** metasoma, dorsal view **F** Scutellum and propodeum, dorsal view.

### Philoplitis striatus

Taxon classificationAnimaliaHymenopteraBraconidae

Fernandez-Triana & Goulet, 2009

Figs 12 , 13 , 16D

Philoplitis striatus Fernandez-Triana & Goulet, 2009: 294, figs 2, 6, 9, 12, 16 (original description).

#### Notes.

This species was described and diagnosed by Fernandez-Triana and Goulet (2009). Here we only provide new images of the species.

**Figure 12.**
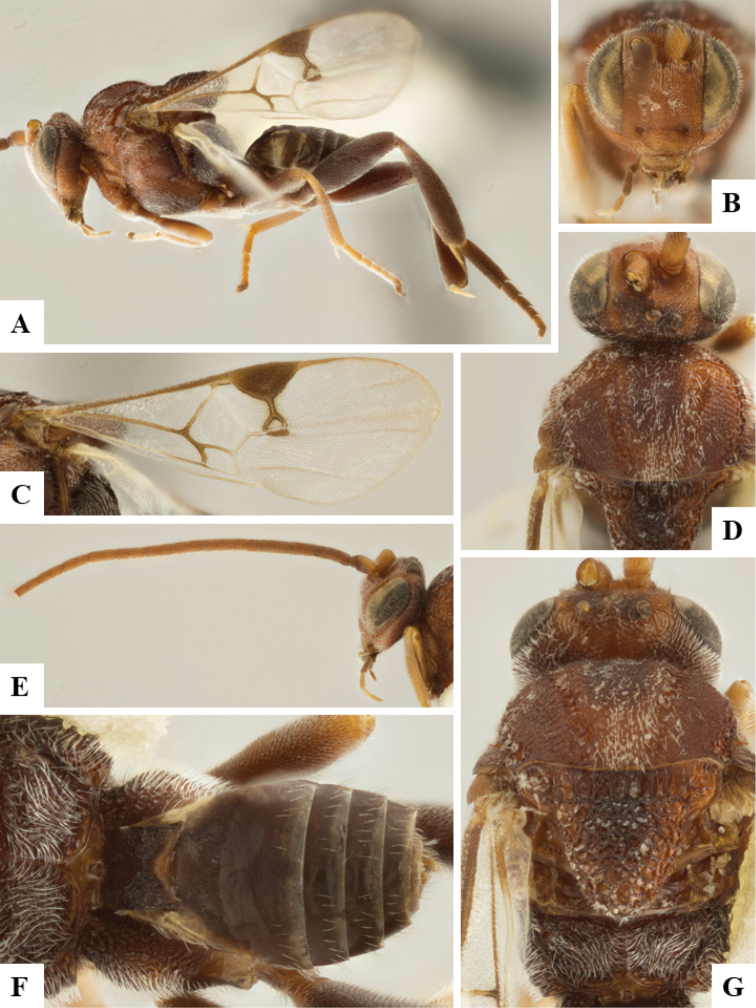
*Philoplitisstriatus*, male holotype **A** habitus, lateral view **B** mesosoma, dorsal view **C** wings **D** head and mesosoma, dorsal view **E** head and antenna, lateral view **F** metasoma, dorsal view **G** head and mesosoma, dorsal view.

**Figure 13.**
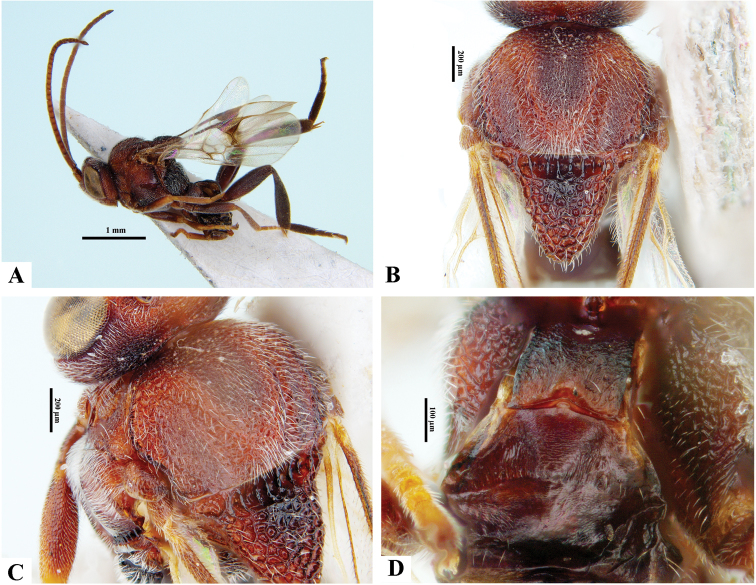
*Philoplitisstriatus*, female **A** habitus, lateral view **B** mesosoma, dorsal view **C** head and mesosoma oblique view **D** metasomal tergites 1-3 (partially), dorsal view.

### Philoplitis trifoveatus

Taxon classificationAnimaliaHymenopteraBraconidae

Ranjith & Fernandez-Triana sp. n.

http://zoobank.org/3B38C228-5D59-426E-AAF8-AD47C3DE7BB0

Figs 14 , 15 , 16C

#### Holotype.

Female, INDIA (DZUC). Holotype locality: Karnataka, MSS SRF, Malaise trap, 1-19.ii.2007.

#### Diagnosis.

*Philoplitistrifoveatus* sp. n. differs from all other species by having the occiput medially with three pits. This new species can be separated from *P.keralensis* sp. n. by having scutellar disc L/W ratio 1.10 ×, and its L 0.90 × that of anteromesoscutum (scutellar disc L/W ratio 1.27×, its length 1.10 × that of anteromesoscutum in *P.keralensis* sp. n.), and by the ratio of fore wing veins r/r-m (2.50 ×in *trifoveatus* sp. n., 2.11 × in *keralensis* sp. n.).

#### Description.

Head distinctly rugose. Frons transversely striate. Occipital carina strongly defined and crenulate. Area centrally between posterior ocelli and occipital carina mostly transversally striate, with only small, shiny spot right above the occipital carina. Antennae longer than body length, L of F2 2.90 × its W, L of F15 2.04× its W. Mesosoma mostly covered with silver setae. Anteromesoscutum coarsely rugose. Notauli deeply impressed, and with impressed postero-lateral area above tegula. Scutellar disc coarsely rugose, apically downward in lateral view. Scutellar disc L/W ratio 1.10 ×, and its L 1.10 × that of anteromesoscutum. Mesopleuron sparsely rugose, but with median, smooth area centrally. Metapleuron rugose. Propodeum rugose with complete median, longitudinal carina. Fore wing ratios, r/3RSa: 1.00 ×; r/r-m: 2.50 ×; r/2RS: 0.80 ×; 3RSa/2M: 1.10 ×; 2RS/2M: 1.30 ×; r-m/2M: 0.44 ×; r/(r-2M): 0.47 ×; height of second sub marginal cell/(r-2M): 0.50 ×. Tarsal claws with one tooth and with arolium subequal to claw length. Metafemur L 3.40 × its maximum width, inner spur of metatibia 0.49 × L of first metatarsomere. T1 faintly rugose laterally and apically, T1 with shallow median longitudinal groove extending beyond half of its length, T1 L 2.35 × its W at posterior margin. T2 smooth, broad, with medial zone outlined by convergent grooves that form a transversely striated triangle, with indistinct carinae markedly divergent, T2 striated medially but without longitudinal striations, T2 medial L 0.37 × its W at posterior margin. T3+ smooth.

**Figure 14.**
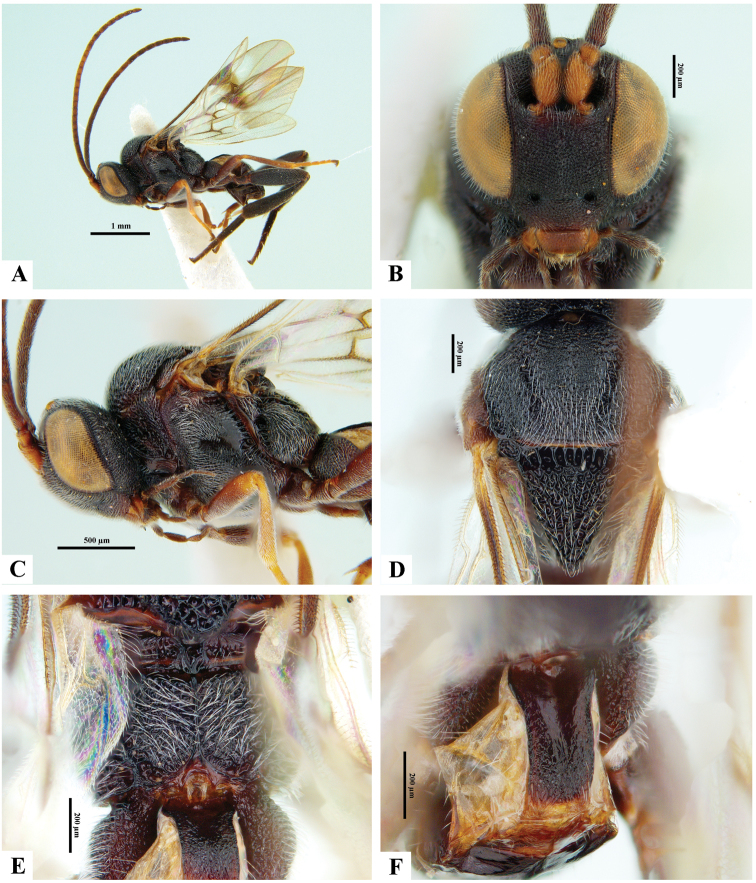
*Philoplitistrifoveatus*, female holotype **A** habitus, lateral view **B** head, frontal view **C** head and mesosoma, lateral view **D** mesosoma, dorsal view **E** propodeum, dorsal view **F** metasomal tergite I, dorsal view.

**Figure 15.**
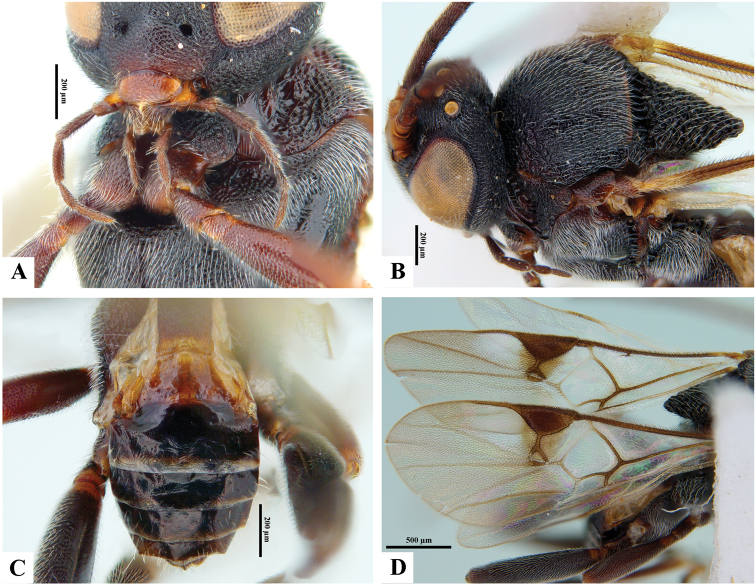
*Philoplitistrifoveatus*, female holotype **A** head and mesosoma, ventral view **B** head and mesosoma, oblique view **C** metasoma, dorsal view **D** wings.

**Figure 16.**
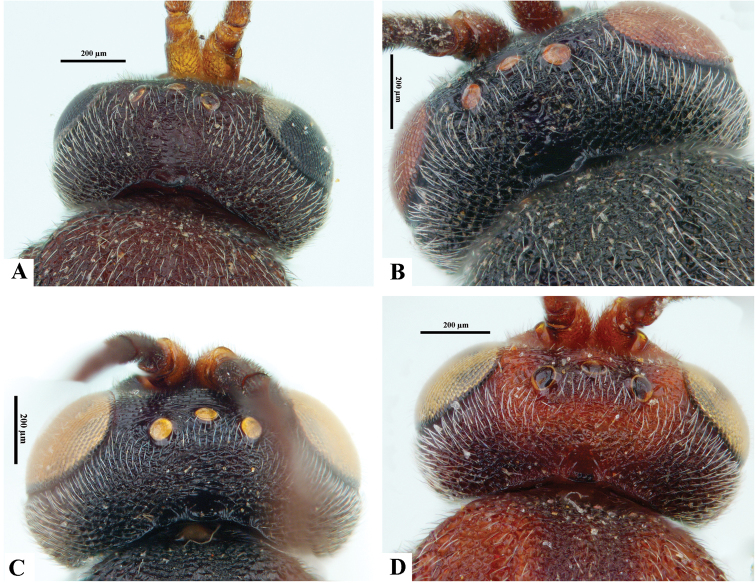
*Philoplitis* spp. from India, dorsal views of heads **A***P.adustipalpus***B***P.keralensis***C***P.trifoveatus***D***P.striatus*.

#### Colour.

Head reddish brown, scape, pedicel and ocelli dark yellow, flagellomeres brown, palpi brown, mesosoma reddish brown, anteromesoscutum dark yellow at posterior margin, profemur mostly brown, yellow apically, protibia and protarsomeres yellow, mesofemur and mesotibia brown, mesotarsomeres brown, metafemur and metatibia black, metatarsomeres brown, metatibial spurs yellow, wing veins and pterostigma light brown with a brownish cloud beneath that extends to vein 2M, metasoma reddish brown except for T1 apically and T2 entirely brownish-yellow, laterotergite 1-3 yellowish-white.

#### Male.

Unknown.

#### Distribution.

India (Karnataka).

#### Etymology.

This species name alludes to the distinctive three pits present in the occiput.

## Supplementary Material

XML Treatment for Philoplitis adustipalpus

XML Treatment for Philoplitis coniferens

XML Treatment for Philoplitis dzangasangha

XML Treatment for Philoplitis keralensis

XML Treatment for Philoplitis margalla

XML Treatment for Philoplitis masneri

XML Treatment for Philoplitis punctatus

XML Treatment for Philoplitis striatus

XML Treatment for Philoplitis trifoveatus

## Supplementary materials

Supplementary material 1 Neighbour Joining Tree (K2P) of available *Philoplitis* sequences (COI) in the Barcode of Life Data System Data type: molecular data This dataset is made available under the Open Database License (http://opendatacommons.org/licenses/odbl/1.0/). The Open Database License (ODbL) is a license agreement intended to allow users to freely share, modify, and use this Dataset while maintaining this same freedom for others, provided that the original source and author(s) are credited.A.P. Ranjith, J. Fernandez-Triana, T. Veena, D.R. Priyadarsanan, M. Nasser
