# Supplementary material for: Four new species of Philoplitis Nixon (Braconidae, Microgastrinae) with an updated key and illustrations of all described species
Source: Zookeys. 2019 May 3;841:125–50. doi: 10.3897/zookeys.841.33549 (PMC6510703; doi:10.3897/zookeys.841.33549)
Supplement: Supplementary material 1 [file zookeys-841-125-s001.pdf]

BOLD TaxonID Tree

Title : Tree Result - Search: Seq Length(10 bp); Tax(Philoplitis); Include public records (7 records returned) (7 records selected)

Date : 30-Jan-2019

Data Type : Nucleotide

Distance Model : Kimura 2 Parameter

Marker : COI-5P

Colourization : [blue]=Stop Codons [red]=Contamination or misidentification

Label : Sample ID

Label : Taxon

Label : Country

Label : Sequence Length

Sequence Count : 7

Species count : 2

Genus count : 1

Family count : 1

Unidentified : 3

BIN Count : 4

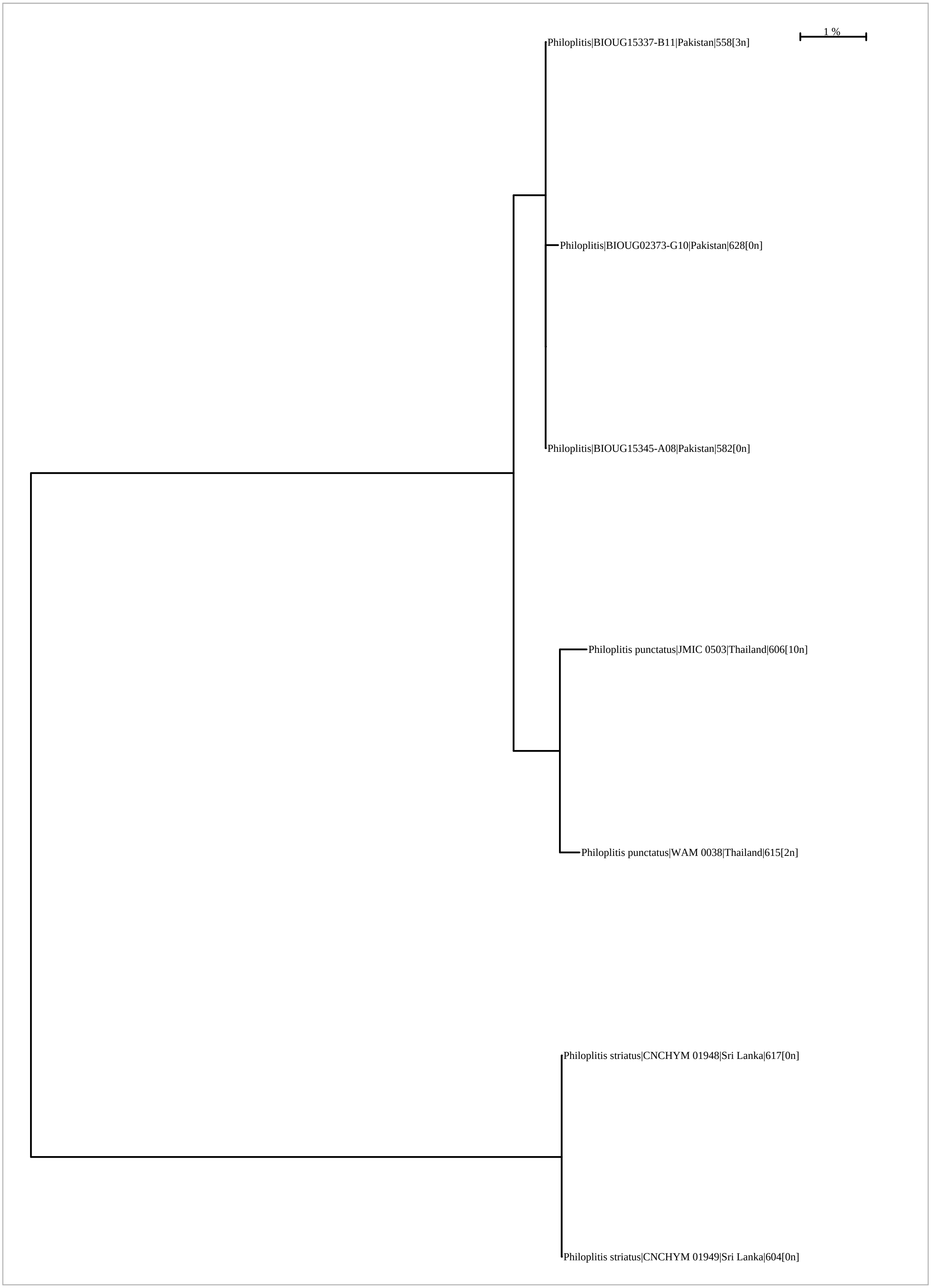
1 %
Philoplitis|BIOUG15337-B11|Pakistan|558[3n]
Philoplitis|BIOUG02373-G10|Pakistan|628[0n]
Philoplitis|BIOUG15345-A08|Pakistan|582[0n]
Philoplitis punctatus|JMIC 0503|Thailand|606[10n]
Philoplitis punctatus|WAM 0038|Thailand|615[2n]
Philoplitis striatus|CNCHYM 01948|Sri Lanka|617[0n]
Philoplitis striatus|CNCHYM 01949|Sri Lanka|604[0n]
